# 2,6-Dichloro-*N*-(4-methyl­phen­yl)benzamide

**DOI:** 10.1107/S1600536812015188

**Published:** 2012-04-18

**Authors:** Peng-Fei Wei, Ling-Yun Hao, Xiao-Li Yang, Yuan-Feng Ye, Zhi-Qiang Feng

**Affiliations:** a College of Materials Engineering, Jinling Institute of Technology, Hongjing Road No.99 Nanjing, Nanjing 211146, People’s Republic of China

## Abstract

In the title compound, C_14_H_11_Cl_2_NO, the two benzene rings are non-coplanar [dihedral angle = 60.9 (3)°]. In the crystal, an amide N—H⋯O hydrogen bond links the mol­ecules into chains which extend along (001).

## Related literature

For the synthesis of the title compound, see: Houlihan *et al.* (1981 ▶). For bond-length data, see: Allen *et al.* (1987 ▶).
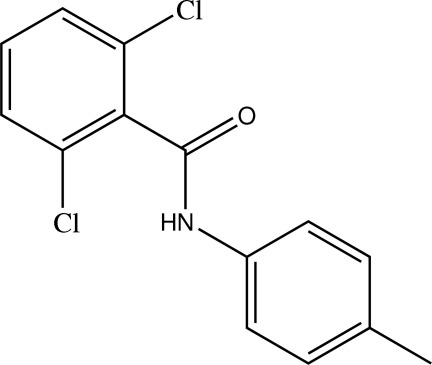


## Experimental

### 

#### Crystal data

C_14_H_11_Cl_2_NO
*M* *_r_* = 280.14
Monoclinic, 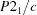

*a* = 11.260 (2) Å
*b* = 12.786 (3) Å
*c* = 9.6700 (19) Å
β = 100.65 (3)°
*V* = 1368.2 (5) Å^3^
*Z* = 4
Mo *K*α radiation
μ = 0.46 mm^−1^
*T* = 293 K
0.30 × 0.10 × 0.10 mm

#### Data collection

Enraf–Nonius CAD-4 diffractometer
Absorption correction: ψ scan (North *et al.*, 1968 ▶) *T*_min_ = 0.874, *T*_max_ = 0.955
2650 measured reflections
2518 independent reflections
1514 reflections with *I* > 2s*I*)
*R*_int_ = 0.033
3 standard reflections every 200 reflections intensity decay: 1%

#### Refinement

*R*[*F* ^2^ > 2σ(*F* ^2^)] = 0.053
*wR*(*F* ^2^) = 0.150
*S* = 1.01
2518 reflections
163 parameters
H-atom parameters constrained
Δρ_max_ = 0.22 e Å^−3^
Δρ_min_ = −0.26 e Å^−3^

### 

Data collection: *CAD-4 Software* (Enraf–Nonius, 1989) ▶; cell refinement: *CAD-4 Software* ▶; data reduction: *XCAD4* (Harms & Wocadlo, 1995 ▶); program(s) used to solve structure: *SHELXS97* (Sheldrick, 2008 ▶); program(s) used to refine structure: *SHELXL97* (Sheldrick, 2008 ▶); molecular graphics: *PLATON* (Spek, 2009 ▶); software used to prepare material for publication: *SHELXL97*.

## Supplementary Material

Crystal structure: contains datablock(s) global, I. DOI: 10.1107/S1600536812015188/zs2197sup1.cif

Structure factors: contains datablock(s) I. DOI: 10.1107/S1600536812015188/zs2197Isup2.hkl

Supplementary material file. DOI: 10.1107/S1600536812015188/zs2197Isup3.cml

Additional supplementary materials:  crystallographic information; 3D view; checkCIF report

## Figures and Tables

**Table 1:** Hydrogen-bond geometry (Å, °)

*D*—H⋯*A*	*D*—H	H⋯*A*	*D*⋯*A*	*D*—H⋯*A*
N—H0*A*⋯O^i^	0.86	1.98	2.839 (4)	173

Symmetry code: (i) 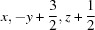.

**Fig. 1.**
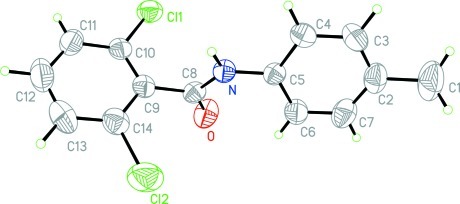
The molecular structure of the title compound showing the atom-numbering scheme, with displacement ellipsoids drawn at the 30% probability level.

**Fig. 2.**
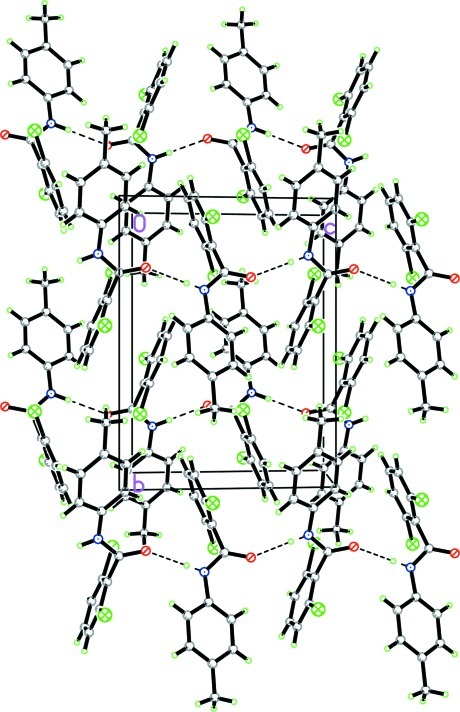
A packing diagram of the title compound viewed down a, with intermolecular hydrogen bonds shown as dashed lines.

## supplementary crystallographic information

### Comment

We report here the crystal structure of the title compound C_14_H_11_Cl_2_NO. In this molecule (Fig. 1), the phenyl and dichlorophenyl rings are non-coplanar [dihedral angle 60.9 (3)°]. In the crystal structure an intermolecular amide N—H···O hydrogen bond (Table 1) links the molecules, giving one-dimensional chains which extend along (001) (Fig. 2).

### Experimental

A mixture of 4-methylbenzenamine (3.2 g, 0.03 mol), 2,6-dichlorobenzoyl chloride (6.3 g, 0.03 mol), and 6 ml of triethylamine in 50 ml of anhydrous tetrahydrofuran was refluxed with stirring for 8 h and then allowed to stand at room temperature. The resulting solids were filtered off and washed with water (2 x 30 mL) then dried, giving 7.2 g of product. Recrystallization from ethanol gave yellow crystals of the title compound. Crystals suitable for X-ray diffraction were obtained by slow evaporation of an ethanol solution.

### Refinement

Hydrogen atoms were positioned geometrically, with C—H = 0.93 Å (aromatic) or 0.96 Å (methyl) and N—H = 0.86 Å and were allowed to ride on their parent atoms, with *U*_iso_(H) = 1.2*U*_eq_(aromatic C, N) or 1.5*U*_eq_(methyl C).

### Crystal data

**Table:**

C_14_H_11_Cl_2_NO	*F*(000) = 576
*M**_r_* = 280.14	*D*_x_ = 1.360 Mg m^−^^3^
Monoclinic, *P*2_1_/*c*	Mo *K*α radiation, λ = 0.71073 Å
Hall symbol: -P 2ybc	Cell parameters from 25 reflections
*a* = 11.260 (2) Å	θ = 9–13°
*b* = 12.786 (3) Å	µ = 0.46 mm^−^^1^
*c* = 9.6700 (19) Å	*T* = 293 K
β = 100.65 (3)°	Block, yellow
*V* = 1368.2 (5) Å^3^	0.30 × 0.10 × 0.10 mm
*Z* = 4	

### Data collection

**Table:**

Enraf–Nonius CAD-4 diffractometer	1514 reflections with *I* > 2s˘*I*)
Radiation source: fine-focus sealed tube	*R*_int_ = 0.033
Graphite monochromator	θ_max_ = 25.4°, θ_min_ = 1.8°
ω/2θ scans	*h* = −13→0
Absorption correction: ψ scan (North *et al.*, 1968)	*k* = 0→15
*T*_min_ = 0.874, *T*_max_ = 0.955	*l* = −11→11
2650 measured reflections	3 standard reflections every 200 reflections
2518 independent reflections	intensity decay: 1%

### Refinement

**Table:**

Refinement on *F*^2^	Primary atom site location: structure-invariant direct methods
Least-squares matrix: full	Secondary atom site location: difference Fourier map
*R*[*F*^2^ > 2σ(*F*^2^)] = 0.053	Hydrogen site location: inferred from neighbouring sites
*wR*(*F*^2^) = 0.150	H-atom parameters constrained
*S* = 1.01	*w* = 1/[σ^2^(*F*_o_^2^) + (0.070*P*)^2^] where *P* = (*F*_o_^2^ + 2*F*_c_^2^)/3
2518 reflections	(Δ/σ)_max_ < 0.001
163 parameters	Δρ_max_ = 0.22 e Å^−^^3^
0 restraints	Δρ_min_ = −0.26 e Å^−^^3^

### Special details

**Table:**

Geometry. All esds (except the esd in the dihedral angle between two l.s. planes) are estimated using the full covariance matrix. The cell esds are taken into account individually in the estimation of esds in distances, angles and torsion angles; correlations between esds in cell parameters are only used when they are defined by crystal symmetry. An approximate (isotropic) treatment of cell esds is used for estimating esds involving l.s. planes.
Refinement. Refinement of F^2^ against ALL reflections. The weighted R-factor wR and goodness of fit S are based on F^2^, conventional R-factors R are based on F, with F set to zero for negative F^2^. The threshold expression of F^2^ > 2sigma(F^2^) is used only for calculating R-factors(gt) etc. and is not relevant to the choice of reflections for refinement. R-factors based on F^2^ are statistically about twice as large as those based on F, and R- factors based on ALL data will be even larger.

### Fractional atomic coordinates and isotropic or equivalent isotropic displacement parameters (Å^2^)

**Table:**

	*x*	*y*	*z*	*U*_iso_*/*U*_eq_	
N	0.2641 (2)	0.8121 (2)	0.1158 (3)	0.0404 (7)	
H0A	0.2757	0.7889	0.2007	0.048*	
O	0.2803 (3)	0.76570 (19)	−0.1064 (2)	0.0660 (8)	
Cl1	0.14387 (9)	0.55959 (8)	0.07524 (10)	0.0635 (3)	
C1	0.0707 (4)	1.2269 (3)	0.0646 (6)	0.0969 (17)	
H1A	0.0283	1.2398	0.1404	0.145*	
H1B	0.0162	1.2352	−0.0236	0.145*	
H1C	0.1361	1.2758	0.0699	0.145*	
Cl2	0.56778 (10)	0.75531 (9)	0.07081 (15)	0.0876 (4)	
C2	0.1203 (3)	1.1167 (3)	0.0760 (5)	0.0601 (11)	
C3	0.1026 (4)	1.0507 (3)	0.1828 (4)	0.0629 (11)	
H3A	0.0596	1.0745	0.2500	0.076*	
C4	0.1474 (3)	0.9499 (3)	0.1924 (4)	0.0533 (9)	
H4A	0.1334	0.9065	0.2649	0.064*	
C5	0.2128 (3)	0.9131 (2)	0.0952 (3)	0.0395 (8)	
C6	0.2314 (3)	0.9779 (3)	−0.0124 (4)	0.0562 (10)	
H6A	0.2750	0.9544	−0.0791	0.067*	
C7	0.1848 (4)	1.0781 (3)	−0.0206 (4)	0.0661 (11)	
H7A	0.1975	1.1210	−0.0941	0.079*	
C8	0.2970 (3)	0.7476 (3)	0.0202 (3)	0.0423 (8)	
C9	0.3616 (3)	0.6510 (3)	0.0813 (3)	0.0418 (8)	
C10	0.3010 (3)	0.5611 (3)	0.1098 (3)	0.0445 (8)	
C11	0.3614 (4)	0.4729 (3)	0.1667 (4)	0.0592 (10)	
H11A	0.3192	0.4132	0.1837	0.071*	
C12	0.4849 (4)	0.4751 (4)	0.1976 (5)	0.0730 (13)	
H12A	0.5265	0.4165	0.2380	0.088*	
C13	0.5485 (4)	0.5612 (4)	0.1707 (4)	0.0710 (12)	
H13A	0.6324	0.5614	0.1921	0.085*	
C14	0.4868 (3)	0.6472 (3)	0.1116 (4)	0.0550 (10)	

### Atomic displacement parameters (Å^2^)

**Table:**

	*U*^11^	*U*^22^	*U*^33^	*U*^12^	*U*^13^	*U*^23^
N	0.0548 (17)	0.0427 (16)	0.0246 (13)	0.0042 (13)	0.0096 (12)	0.0009 (12)
O	0.112 (2)	0.0577 (16)	0.0291 (13)	0.0081 (15)	0.0157 (13)	−0.0012 (12)
Cl1	0.0531 (6)	0.0723 (7)	0.0669 (7)	−0.0039 (5)	0.0156 (5)	0.0100 (5)
C1	0.092 (4)	0.054 (3)	0.139 (5)	0.020 (3)	0.007 (3)	0.002 (3)
Cl2	0.0715 (8)	0.0718 (8)	0.1247 (11)	−0.0208 (6)	0.0314 (7)	−0.0195 (7)
C2	0.049 (2)	0.044 (2)	0.082 (3)	0.0040 (18)	0.000 (2)	−0.003 (2)
C3	0.062 (3)	0.060 (3)	0.072 (3)	0.008 (2)	0.027 (2)	−0.010 (2)
C4	0.060 (2)	0.054 (2)	0.050 (2)	0.0024 (19)	0.0204 (18)	−0.0009 (18)
C5	0.0430 (18)	0.0413 (19)	0.0337 (17)	−0.0001 (15)	0.0059 (14)	0.0015 (15)
C6	0.074 (3)	0.052 (2)	0.047 (2)	0.009 (2)	0.0222 (19)	0.0045 (18)
C7	0.083 (3)	0.051 (2)	0.063 (3)	0.003 (2)	0.010 (2)	0.013 (2)
C8	0.051 (2)	0.0449 (19)	0.0318 (18)	−0.0029 (16)	0.0087 (15)	0.0000 (16)
C9	0.050 (2)	0.045 (2)	0.0323 (17)	0.0039 (16)	0.0115 (15)	−0.0071 (15)
C10	0.050 (2)	0.051 (2)	0.0338 (17)	0.0035 (18)	0.0131 (15)	−0.0029 (17)
C11	0.072 (3)	0.051 (2)	0.057 (2)	0.008 (2)	0.018 (2)	0.0078 (18)
C12	0.073 (3)	0.067 (3)	0.077 (3)	0.027 (3)	0.011 (2)	0.009 (2)
C13	0.048 (2)	0.086 (3)	0.076 (3)	0.016 (2)	0.002 (2)	−0.004 (3)
C14	0.054 (2)	0.053 (2)	0.060 (2)	−0.0002 (19)	0.0143 (18)	−0.0102 (19)

### Geometric parameters (Å, º)

**Table:**

N—C8	1.341 (4)	C4—H4A	0.9300
N—C5	1.414 (4)	C5—C6	1.376 (4)
N—H0A	0.8600	C6—C7	1.381 (5)
O—C8	1.225 (4)	C6—H6A	0.9300
Cl1—C10	1.739 (4)	C7—H7A	0.9300
C1—C2	1.513 (5)	C8—C9	1.498 (5)
C1—H1A	0.9600	C9—C14	1.387 (5)
C1—H1B	0.9600	C9—C10	1.390 (5)
C1—H1C	0.9600	C10—C11	1.379 (5)
Cl2—C14	1.740 (4)	C11—C12	1.367 (6)
C2—C7	1.376 (5)	C11—H11A	0.9300
C2—C3	1.377 (5)	C12—C13	1.365 (6)
C3—C4	1.381 (5)	C12—H12A	0.9300
C3—H3A	0.9300	C13—C14	1.368 (5)
C4—C5	1.379 (4)	C13—H13A	0.9300
			
C8—N—C5	128.5 (3)	C2—C7—C6	122.4 (4)
C8—N—H0A	115.8	C2—C7—H7A	118.8
C5—N—H0A	115.8	C6—C7—H7A	118.8
C2—C1—H1A	109.5	O—C8—N	124.2 (3)
C2—C1—H1B	109.5	O—C8—C9	121.6 (3)
H1A—C1—H1B	109.5	N—C8—C9	114.2 (3)
C2—C1—H1C	109.5	C14—C9—C10	116.5 (3)
H1A—C1—H1C	109.5	C14—C9—C8	120.8 (3)
H1B—C1—H1C	109.5	C10—C9—C8	122.7 (3)
C7—C2—C3	117.2 (4)	C11—C10—C9	122.1 (3)
C7—C2—C1	121.3 (4)	C11—C10—Cl1	118.5 (3)
C3—C2—C1	121.5 (4)	C9—C10—Cl1	119.4 (3)
C2—C3—C4	121.4 (4)	C12—C11—C10	118.5 (4)
C2—C3—H3A	119.3	C12—C11—H11A	120.7
C4—C3—H3A	119.3	C10—C11—H11A	120.7
C5—C4—C3	120.5 (3)	C13—C12—C11	121.5 (4)
C5—C4—H4A	119.8	C13—C12—H12A	119.2
C3—C4—H4A	119.8	C11—C12—H12A	119.2
C6—C5—C4	119.0 (3)	C12—C13—C14	119.0 (4)
C6—C5—N	122.8 (3)	C12—C13—H13A	120.5
C4—C5—N	118.1 (3)	C14—C13—H13A	120.5
C5—C6—C7	119.5 (3)	C13—C14—C9	122.3 (4)
C5—C6—H6A	120.2	C13—C14—Cl2	119.1 (3)
C7—C6—H6A	120.2	C9—C14—Cl2	118.6 (3)
			
C7—C2—C3—C4	−0.2 (6)	O—C8—C9—C10	−96.5 (4)
C1—C2—C3—C4	179.6 (4)	N—C8—C9—C10	85.6 (4)
C2—C3—C4—C5	0.8 (6)	C14—C9—C10—C11	0.7 (5)
C3—C4—C5—C6	−0.8 (5)	C8—C9—C10—C11	−179.7 (3)
C3—C4—C5—N	175.4 (3)	C14—C9—C10—Cl1	179.8 (2)
C8—N—C5—C6	−26.7 (5)	C8—C9—C10—Cl1	−0.5 (4)
C8—N—C5—C4	157.2 (3)	C9—C10—C11—C12	1.0 (5)
C4—C5—C6—C7	0.2 (5)	Cl1—C10—C11—C12	−178.2 (3)
N—C5—C6—C7	−175.8 (3)	C10—C11—C12—C13	−1.4 (6)
C3—C2—C7—C6	−0.4 (6)	C11—C12—C13—C14	0.2 (7)
C1—C2—C7—C6	179.8 (4)	C12—C13—C14—C9	1.6 (6)
C5—C6—C7—C2	0.4 (6)	C12—C13—C14—Cl2	−177.5 (3)
C5—N—C8—O	−4.5 (5)	C10—C9—C14—C13	−2.0 (5)
C5—N—C8—C9	173.4 (3)	C8—C9—C14—C13	178.4 (3)
O—C8—C9—C14	83.1 (4)	C10—C9—C14—Cl2	177.1 (2)
N—C8—C9—C14	−94.8 (4)	C8—C9—C14—Cl2	−2.5 (4)

### Hydrogen-bond geometry (Å, º)

**Table:**

*D*—H···*A*	*D*—H	H···*A*	*D*···*A*	*D*—H···*A*
N—H0*A*···O^i^	0.86	1.98	2.839 (4)	173

Symmetry code: (i) *x*, −*y*+3/2, *z*+1/2.
